# Establishment of an MRI-based radiomics model for distinguishing between intramedullary spinal cord tumor and tumefactive demyelinating lesion

**DOI:** 10.1186/s12880-024-01499-8

**Published:** 2024-11-21

**Authors:** Zifeng Zhang, Ning Li, Yuhang Qian, Huilin Cheng

**Affiliations:** 1 School of Medicine, Southeast University, Nanjing, China; 2 Department of Neurosurgery, Affiliated Zhongda Hospital, Southeast University, Nanjing, China

**Keywords:** Intramedullary spinal cord tumor, Tumefactive demyelinating lesion, Magnetic resonance images, Radiomics

## Abstract

**Objective:**

Differentiating intramedullary spinal cord tumor (IMSCT) from spinal cord tumefactive demyelinating lesion (scTDL) remains challenging with standard diagnostic approaches. This study aims to develop and evaluate the effectiveness of a magnetic resonance imaging (MRI)-based radiomics model for distinguishing scTDL from IMSCT before treatment initiation.

**Methods:**

A total of 75 patients were analyzed in this retrospective study, comprising 55 with IMSCT and 20 with scTDL. Radiomics features were extracted from T1- and T2-weighted imaging (T1&T2WI) scans upon admission. Ten classification algorithms were employed: logistic regression (LR); naive bayes (NaiveBayes); support vector machine (SVM); k nearest neighbors (KNN); random forest (RF); extra trees (ExtraTrees); eXtreme gradient boosting (XGBoost); light gradient boosting machine (LightGBM); gradient boosting (GradientBoosting); and multi-Layer perceptron (MLP). The performance of the optimal model was then compared to radiologists' assessments.

**Results:**

This study developed 30 predictive models using ten classifiers across two imaging sequences. The MLP model with two sequences (T1&T2WI) emerged as the most effective one, showing superior accuracy in MRI analysis with an area under the curve (AUC) of 0.991 in training and 0.962 in testing. Moreover, statistical analyses highlighted the radiomics model significantly outperformed radiologists' assessments (*p* < 0.05) in distinguishing between IMSCT and scTDL.

**Conclusion:**

We present an MRI-based radiomics model with high diagnostic accuracy in differentiating IMSCT from scTDL. The model’s performance was comparable to junior radiologists, highlighting its potential as an effective diagnostic aid in clinical practice.

**Supplementary Information:**

The online version contains supplementary material available at 10.1186/s12880-024-01499-8.

## Introduction

Primary spinal cord tumors account for 2%–4% of all central nervous system malignancies [1, 2]. Among these, intramedullary spinal cord tumor (IMSCT) comprises 20%–30% of primary spinal cord tumors, with astrocytoma and ependymoma representing the predominant types [3]. These tumors commonly occur in the cervical or thoracic spine [4, 5] and typically present with symptoms such as back or radicular pain, followed by neurological deficits like motor weakness or sensory disturbances [6]. Accurate preoperative evaluation is crucial in formulating treatment strategies for IMSCT, with surgical gross total resection often being the primary approach [7]. Differentiating IMSCTs from other intramedullary lesions, such as spinal cord tumefactive demyelinating lesion (scTDL), can be challenging [8–10]. scTDL, initially described by Van der Velden et al., is identified by predominantly white matter areas exceeding 2 cm [8, 11]. The rarity of spinal cord involvement cases has limited detailed clinical and radiographic documentation [12]. The treatment for scTDL differs significantly from that for IMSCT, with corticosteroids and symptom-relieving medications primarily administered [13].

Magnetic resonance imaging (MRI) stands as the first-line method for diagnosing IMSCT and scTDL. IMSCT generally appears as isointense to hypointense on T1-weighted images and hyperintense on T2-weighted images [14], while scTDL often displays similar imaging characteristics and clinical presentations [12], contributing to a high risk of misdiagnosis between the two conditions. Current diagnostic methods for scTDL, including cerebrospinal fluid (CSF) analysis, are typically invasive, highlighting the need for a non-invasive alternative to enhance diagnostic accuracy.

Radiomics, an emerging field, utilizes a wide range of imaging features invisible through traditional assessments, such as texture, intensity, heterogeneity, and shape, which reflect cellular-level variations within lesions [15–17]. Previous studies have employed radiomics models for the preoperative prediction of central system conditions like glioma and meningioma [18, 19], and non-central system diseases including breast, colorectal, bladder cancer, and lung adenocarcinoma [20–23], indicating its potential application in clinical diagnosis. However, machine learning and radiomics applications in predicting differential diagnoses between IMSCT and scTDL remains underexplored. Therefore, this study aims to develop an effective preoperative predictive model leveraging radiomic features to enhance diagnostic of these two lesions.

## Materials and methods

### Study population

**Fig. 1:**
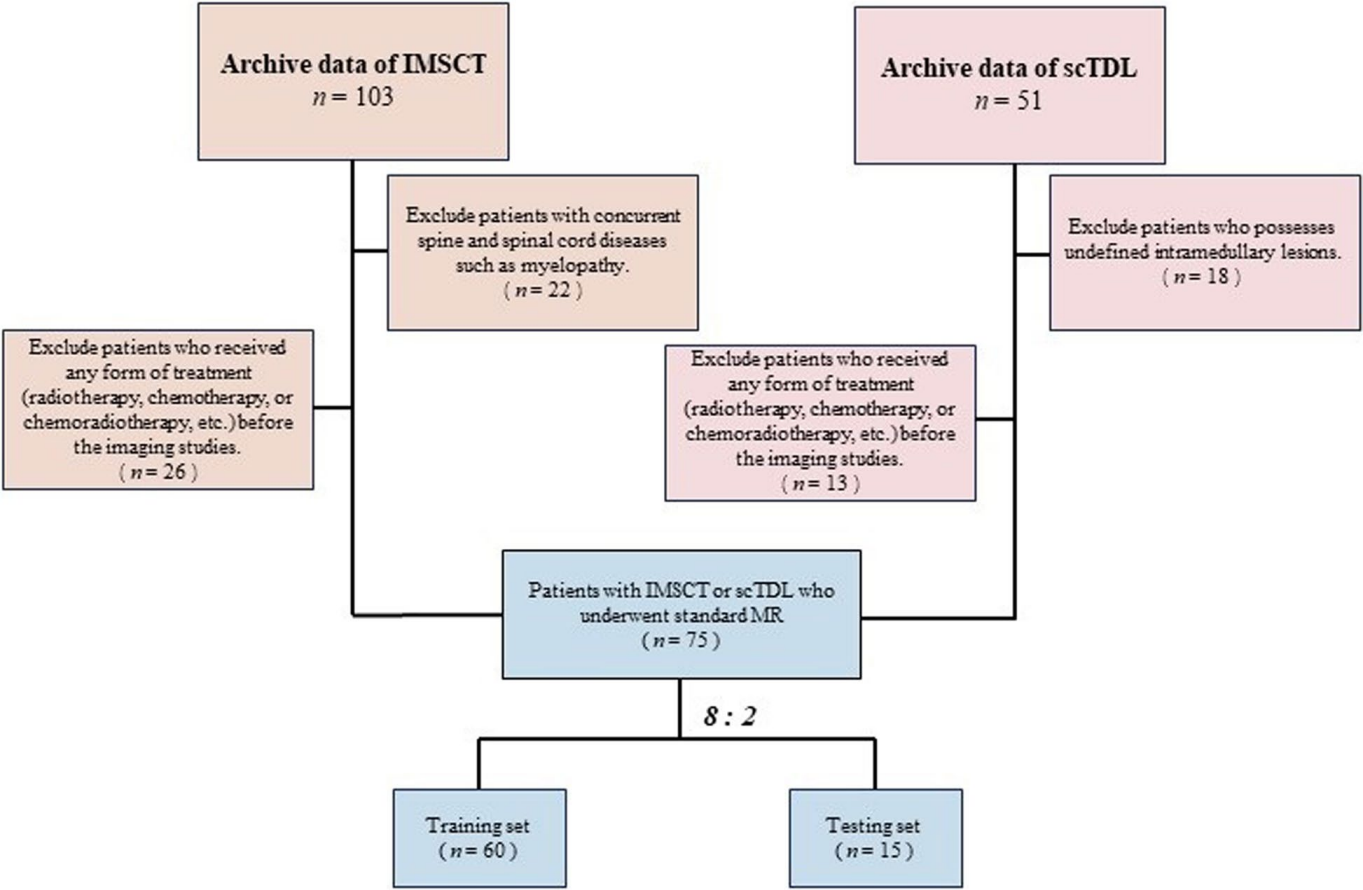
Flow chart demonstrating the inclusion and exclusion criteria for the study participants with IMSCT and scTDL

This analysis included 75 patient records from October 2018 to August 2023. Ethical approval is granted by the Ethics Committee of Southeast University (approval number: 2023ZDSYLL446-P01). Patients' informed consent was waived due to the utilization of anonymized data (Fig. 1).

For IMSCT patients, the inclusion criteria were those: 1) with pathological diagnosis of IMSCT, 2) had pre-treatment MRI data upon admission (within 10 days). The exclusion criteria were those: 1) with concurrent spine and spinal cord diseases such as myelopathy, 2) received any form of treatment (radiotherapy, chemotherapy, or chemoradiotherapy, etc.) before the imaging studies.

For scTDL patients, the inclusion criteria include those: 1) with both radiological evidence and cerebrospinal fluid test results (AQP4-IgG positive or the presence of oligoclonal bands), 2) had pre-treatment MRI data upon admission (within 10 days). The exclusion criteria were patients who 1) possesses undefined intramedullary lesions, 2) received any form of treatment (radiotherapy, chemotherapy, or chemoradiotherapy, etc.) before the imaging studies.

### MRI Scanning

MRI images were obtained using 3.0 T scanners (Siemens MAGNETOM Verio 3.0 T, or Philips Ingenia 3.0 T). Both T1- and T2-weighed images were acquired in sagittal, axial, and coronal sections. Supplementary Table 1 shows the parameters of the selected sequences of each MRI scanner.

### Image segmentation

**Fig. 2:**
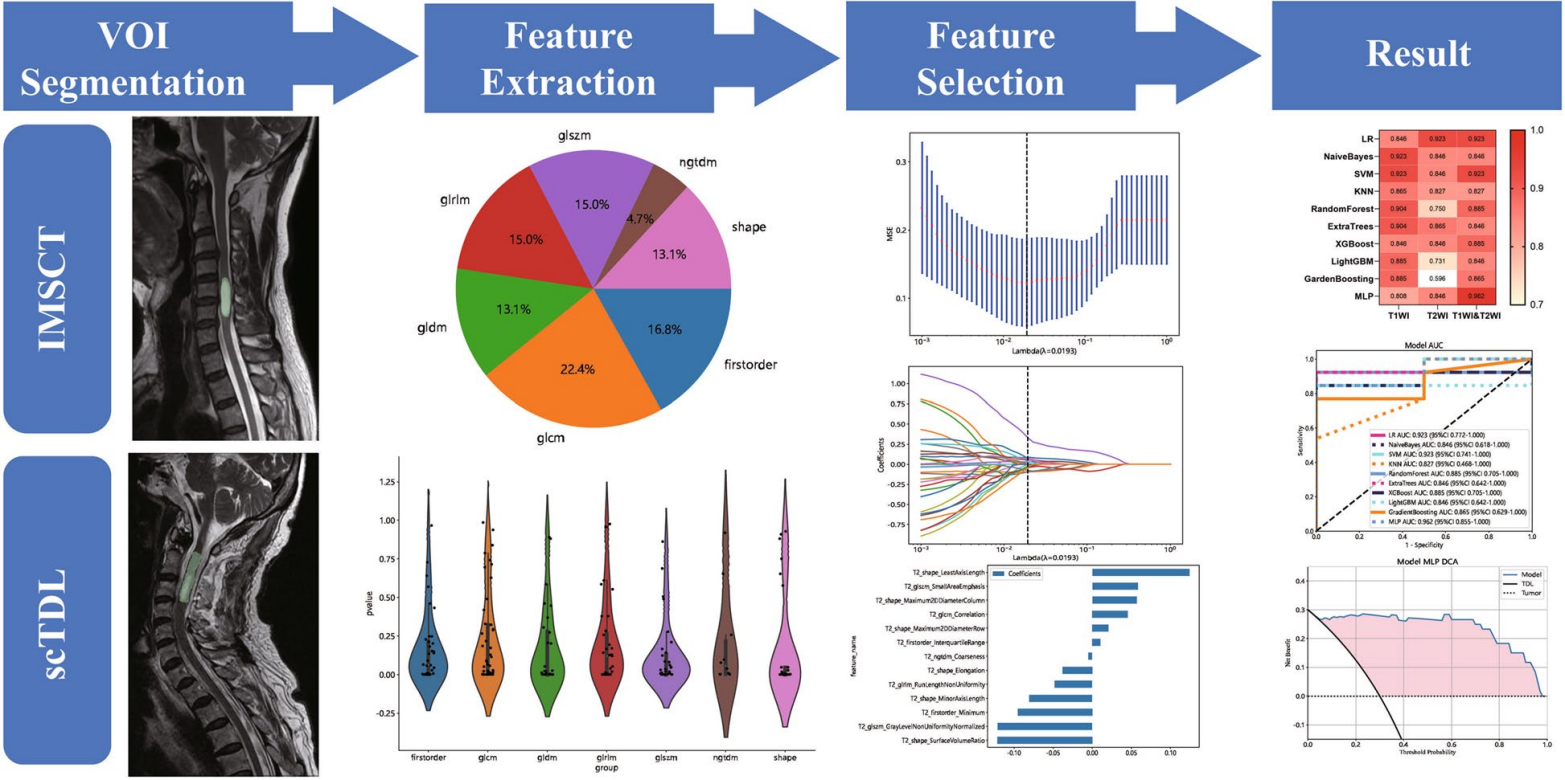
Radiomics workflow

The workflow of the study is shown in Fig. 2. We accessed DICOM format images of axial T1WI and T2WI for each case on the Picture Archiving and Communication System (PACS). Subsequent manual segmentations of the volume of interest (VOI) were carried out utilizing 3D Slicer software (HTTP:// https://www.slicer.org; version 5.0.3). The target of image segmentation is the lesion area. When there were multiple lesions in the spinal cord, the largest lesion was chosen.

Two radiologists (Rad. A and B) defined the VOI independently in a blinded manner. VOI delineation was repeated 2 months later to evaluate the intra-observer reliability. The intraclass correlation coefficient (ICC) was calculated for each feature to assess inter-observer consistency and intra-observer reliability. Cases with an ICC below 0.75 were omitted from further analysis.

### Data preprocessing

The dataset was randomly divided into the training and testing sets in an 8:2 ratio. The training dataset included all cases for model development, while the testing dataset was used for external validation of the model's performance.

Voxel spacing refers to the physical distance separating two adjacent voxels within an image. Medical imaging data often present variability in voxel spacing, attributed to the use of different scanners or imaging protocols. To counteract the variability in voxel spacing, spatial normalization techniques are commonly applied. In our study, we employed a fixed-resolution resampling technique to overcome these spatial challenges. Through resampling, all images were adjusted to a uniform voxel spacing of 3 mm. Subsequently, image data underwent z-score standardization for intensity normalization, ensuring a mean of zero and a standard deviation of one.

### Radiomics feature extraction

The handcrafted features in our analysis are categorized into three main types: 1) geometric, involving the lesion's three-dimensional shape and structure; 2) intensity, detailing the distribution of voxel intensities across the tumor and offering insights into the intensity patterns; and 3) texture, which delves into a more sophisticated level of spatial arrangement of voxel intensities, elucidating complex patterns and interrelationships.

To capture detailed texture information, we employed advanced techniques such as the Gray Level Co-occurrence Matrix (GLCM), Gray Level Run Length Matrix (GLRLM), Gray Level Size Zone Matrix (GLSZM), and the Neighboring Gray Tone Difference Matrix (NGTDM). We identified a total of 107 unique handcrafted features, including 18 focused on shape, 14 on first-order statistics, and 75 on texture characteristics. The extraction of these features was executed through the PyRadiomics platform (available at http://pyradiomics.readthedocs.io).

### Radiomics feature selection

An independent sample T-test and univariate feature screening were applied to all radiomics features. For multiple comparisons, the Bonferroni correction method was applied to adjust the significance threshold, with only those features demonstrating an adjusted *p* < 0.05 being considered significant. To reduce multicollinearity, Pearson's correlation analysis was conducted, and features exhibiting a Pearson's r value greater than 0.9 between any pair of features were removed.

In the process of feature selection, a strategy of greedy recursive elimination was implemented. This method progressively removes the most redundant feature in the set during each cycle. Additionally, the Least Absolute Shrinkage and Selection Operator (LASSO) technique was employed for multivariable feature selection, aiding in identifying a more impactful subset of features for developing the classification model. The LASSO's optimization goal can be described by the formula:

$$y=\left(\frac{1}{2n}\right){|\left|y-\omega X\right||}^{2} + \alpha {|\left|\omega \right||}_{1},$$

where n represents the sample size, α is a predefined constant, and ${|\left|\omega \right||}_{1}$ denotes the L1-norm of the coefficient vector.

### Radiomics model construction

Models based on individual MRI sequences (T1WI or T2WI) as well as a dual-sequence model were developed using ten different classification algorithms. The performance of the single-sequence models was compared to the combined model. Receiver Operating Characteristic (ROC) curves for both the training and validation groups were generated for both the training and testing groups to assess predictive accuracy. For each group, we computed the mean values of the Area Under the ROC Curve (AUC), as well as the accuracy, F1-score, sensitivity, and specificity metrics. Furthermore, to ascertain the clinical applicability of these models, we conducted analyses using calibration curves and decision curve analysis (DCA) for each model.

### Radiologists' diagnoses

Two junior radiologists (Rad. C and D), each with diagnostic experience under 5 years made the radiological diagnosis for all cases independently in a blinded manner. Then, we employed the best radiomics model for comparison with the radiologists' models. We assessed the discriminative prowess of the radiologists and the radiomics model using the Delong test. A *p* value less than 0.05 signified statistical significance. We gauged the inter-reader consensus between the radiologists via the Cohen kappa test. An excellent agreement was represented by a kappa value within the range of 0.81–1.00, a good agreement within 0.61–0.80, a moderate agreement in the span of 0.41–0.60, while a fair agreement fell between 0.21–0.4, and poor agreement ranged from 0 to 0.2.

### Statistical analysis

SPSS software (version 26.0, IBM, USA) was used for statistical analysis. Results were presented as mean ± standard deviation or median ± quartiles (conforms to a normal distribution). For comparisons between groups, we utilized the T test for continuous variables and the Chi-square test for categorical variables. A statistical significance was established with a *p* value less than 0.05.

## Results

### Patient characteristics

A total of 75 patients were included, of whom 55 had IMSCT (mean age 50.3 years) and 20 had scTDL (mean age 50.0 years). The scTDL group included patients diagnosed with multiple sclerosis (MS) accounting for 55.0% (*n* = 11), and neuromyelitis optica (NMO) accounting for 45.0% (*n* = 9). The IMSCT group exclusively comprised patients diagnosed with ependymoma (EPN) representing 87.3% (*n* = 48) and astrocytoma (AST) representing 12.7% (*n* = 7). The scTDL group had lesions predominantly in the cervical spinal cord (75.0%, *n* = 15), with a minority in the thoracic spinal cord (25.0%, *n* = 5). In contrast, lesions in the IMSCT group were more widely distributed with 54.6% (*n* = 30) in the cervical spinal cord, 23.6% (*n* = 13) in the thoracic spinal cord, and 21.8% (*n* = 12) in the lumbar spinal cord. Data is presented in Table 1.

**Table 1:** Characteristics of the enrolled patients (*n* = 55)

	scTDL	IMSCT	*P* value
Age	49.95 ± 16.44	50.31 ± 15.57	0.671
Gender			
Male(%)	7 (35.00)	29 (52.73)	0.170
Female(%)	13 (65.00)	26 (47.27)	
Diagnosis			
AST(%)	/	7 (12.73)	
EPN(%)	/	48 (87.27)	
MS(%)	11 (55.00)	/	
NMO(%)	9 (45.00)	/	
Location			
Cervical(%)	15 (75.00)	30 (54.55)	0.000
Thoracic(%)	5 (25.00)	13 (23.64)	
Lumbar(%)	0 (0.00)	12 (21.82)	

Student's t-test for the normally distributed continuous variable (age) (conforms to a normal distribution) and Pearson chi-square test for the categorical variables (gender)

*AST* Astrocytoma, *EPN* Ependymoma, *MS* Multiple Sclerosis, *NMO* Neuromyelitis Optica

### The MLP two-sequence model as the most efficient prediction model

**Fig. 3:**
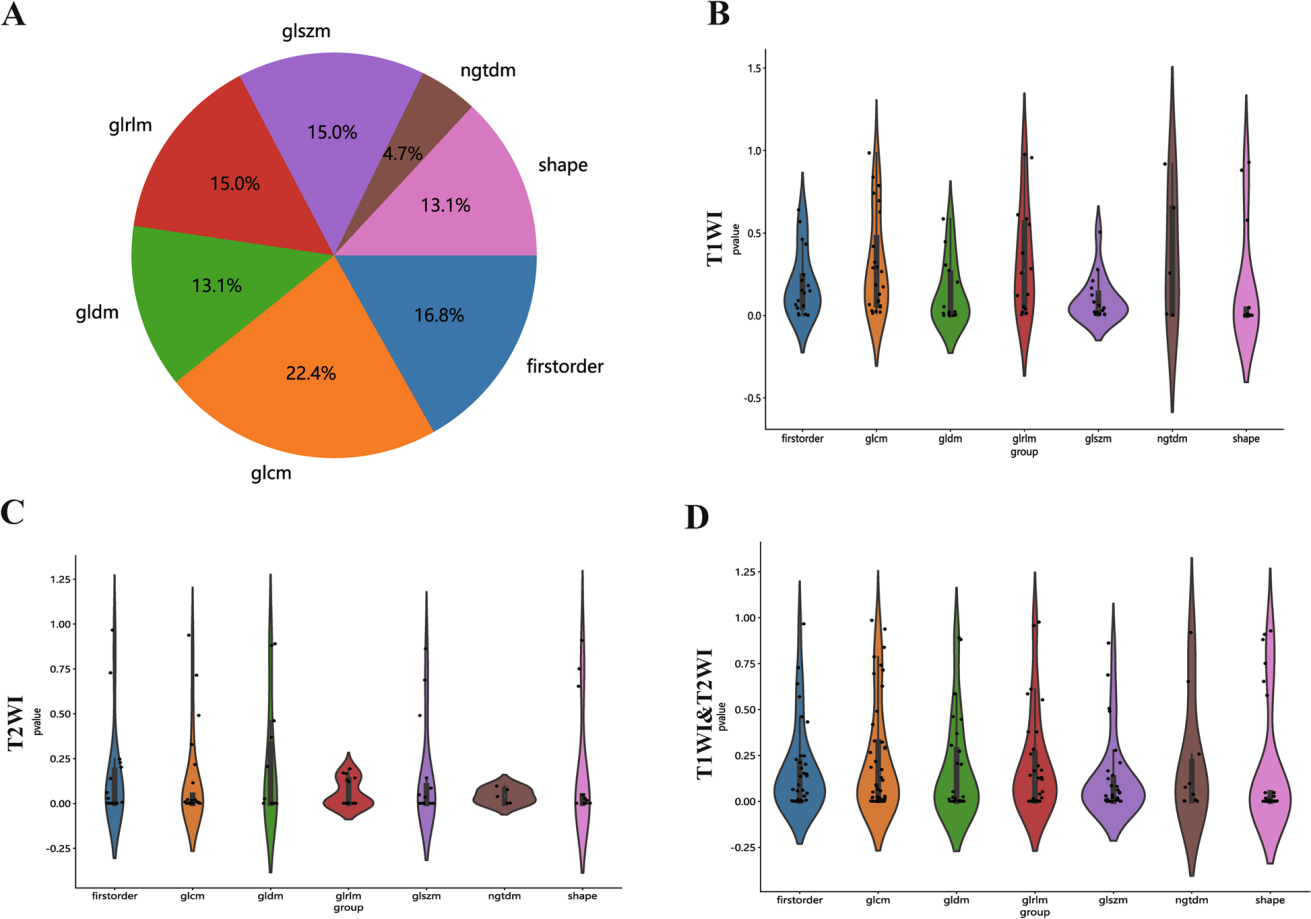
The proportion, distribution and *p* values of various radiomics features

**Fig. 4:**
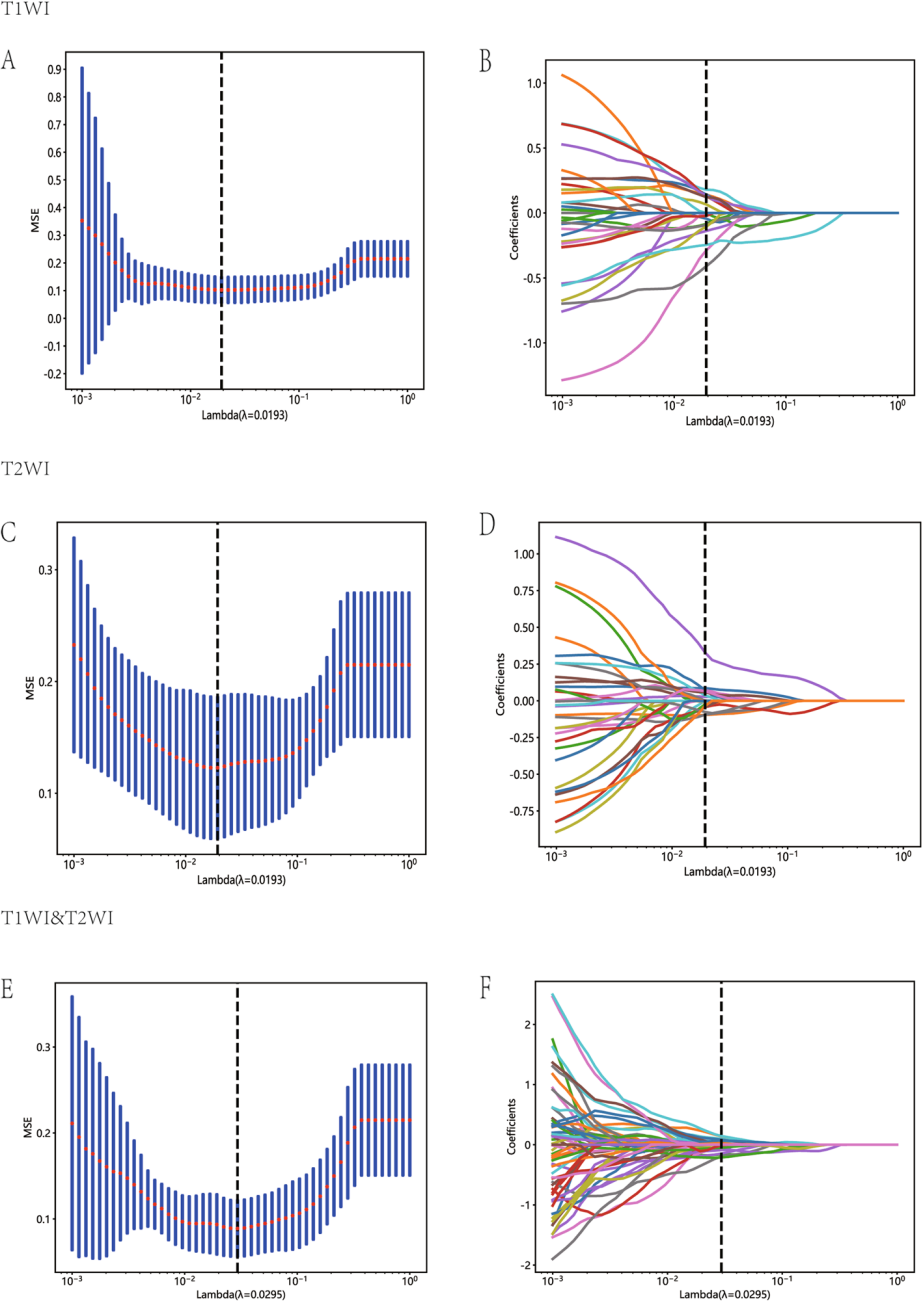
Diagram of the feature selection process. Area under the curve under different number of features in the least absolute shrinkage and selection operator fitting process (**A**, **C** and **E**). Diagram of the characteristic and coefficient change under different α parameters for the three models (**B**, **D** and **F**). (Top: T1WI; Middle: T2WI; Bottom: T1&T2WI)

Radiomic features demonstrating an ICC of ≥ 0.85 were deemed to have high reliability both within and between raters. Spearman's correlation coefficients were calculated to assess multicollinearity, retaining only features with low inter-correlations (in Supplementary Table 2). Figure 3 illustrates the ICC values along with their corresponding *p* values for these features. The LASSO algorithm was subsequently applied to determine optimal the best feature set for T1, T2, and the combined datasets (Fig. 4).

**Fig. 5:**
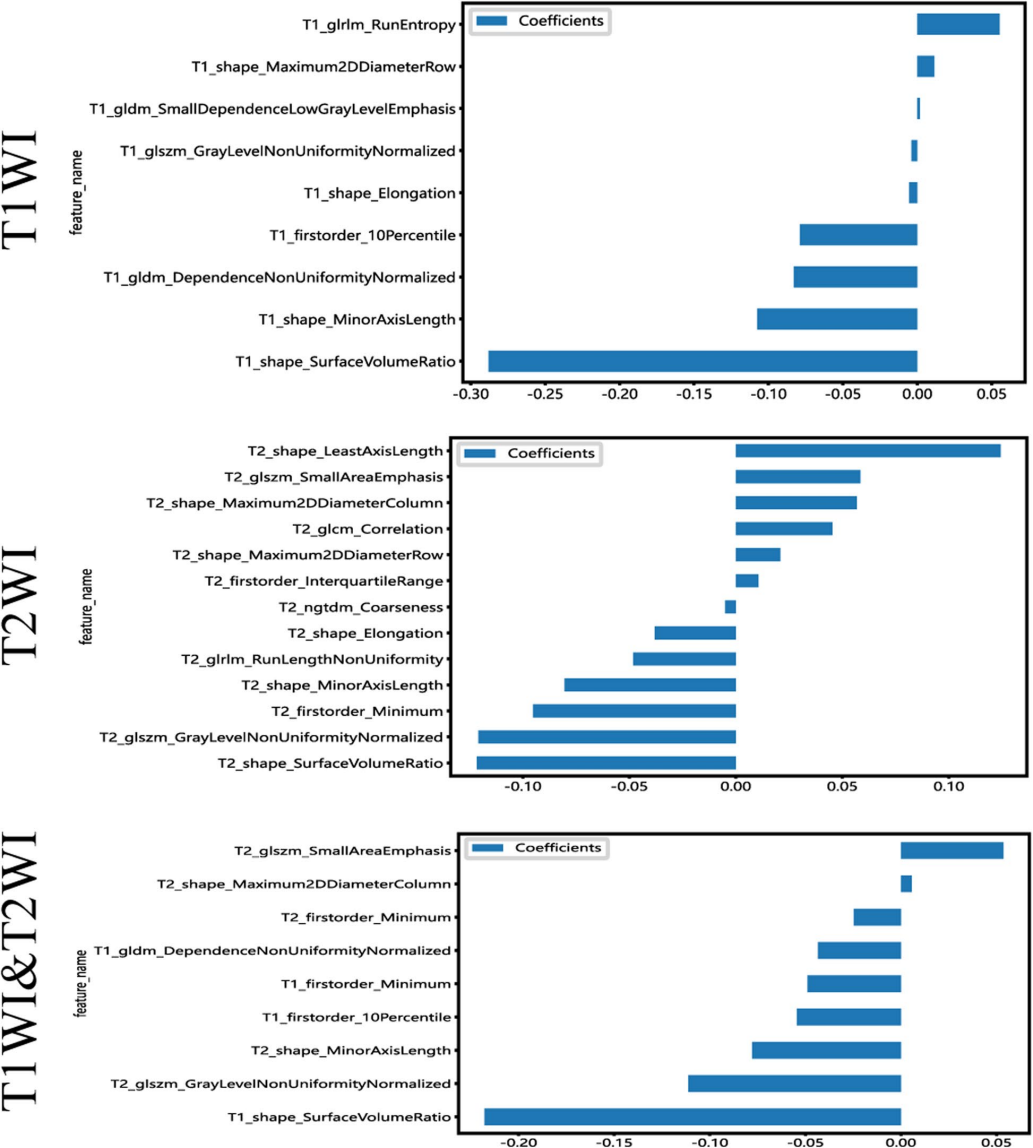
Bar chart showing the selected features in corresponding coefficients, as identified by Lasso Regression

The features with the non-zero coefficient value were retained, including two first-order statistical features, three 3D shape features, two GLSZM features, and one GLDM feature (first order minimum in common, Fig. 5). The radiomics signature was then established based on the these chosen features.

**Fig. 6:**
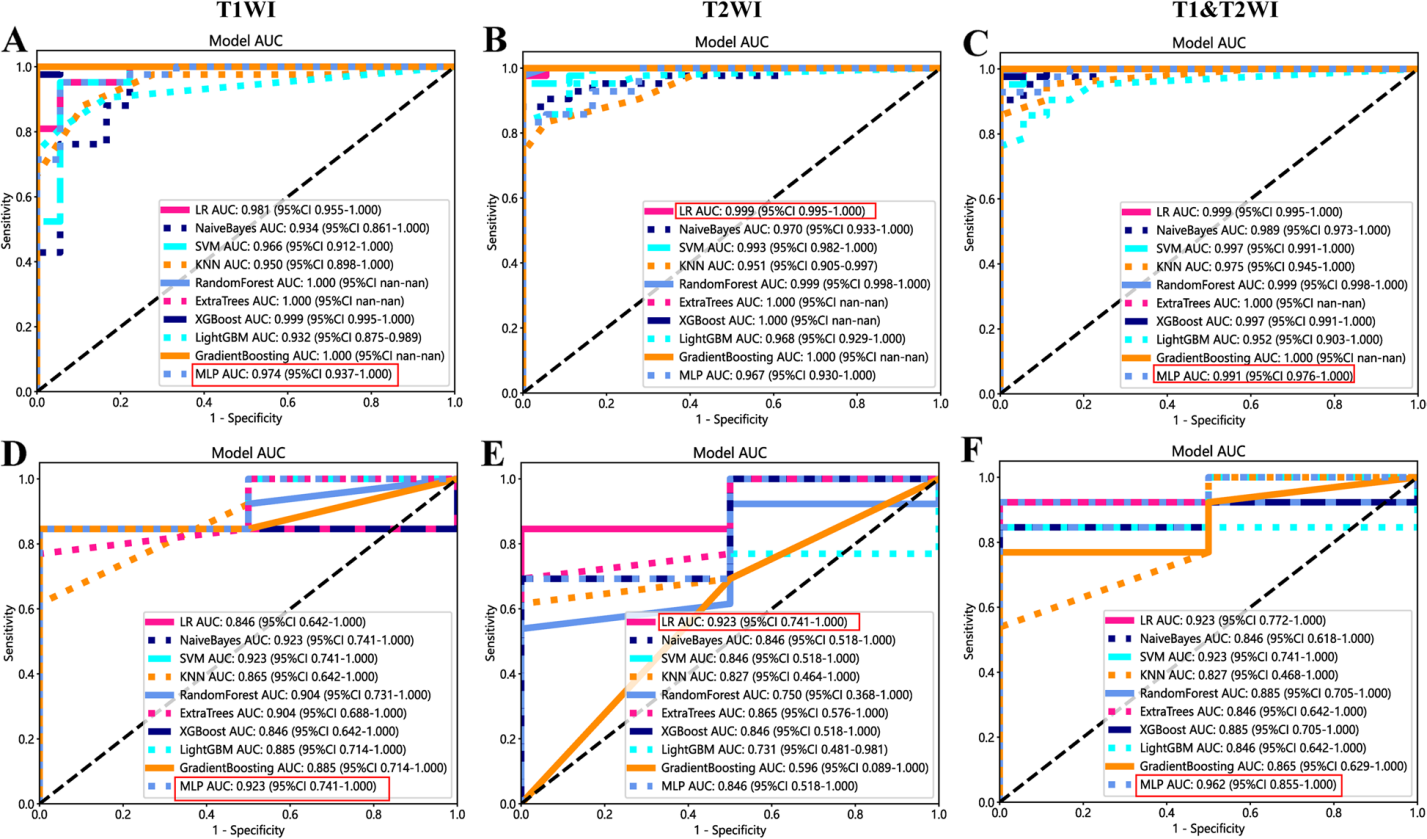
Receiver operating characteristic curves of different diagnostic models in the training set (**A**, **B** and **C**) and testing set (**D**, **E** and **F**). T1WI (left), T2WI (middle), and T1&T2WI (right). The combined radiomics model of the primary tumor with a 10-mm peritumoral extension (CRprim + 10) reached the highest AUC of 0.995 (95% CI, 0.991–0.999) in the training set and 0.872 (95% CI, 0.847–0.897) in the testing set

**Fig. 7:**
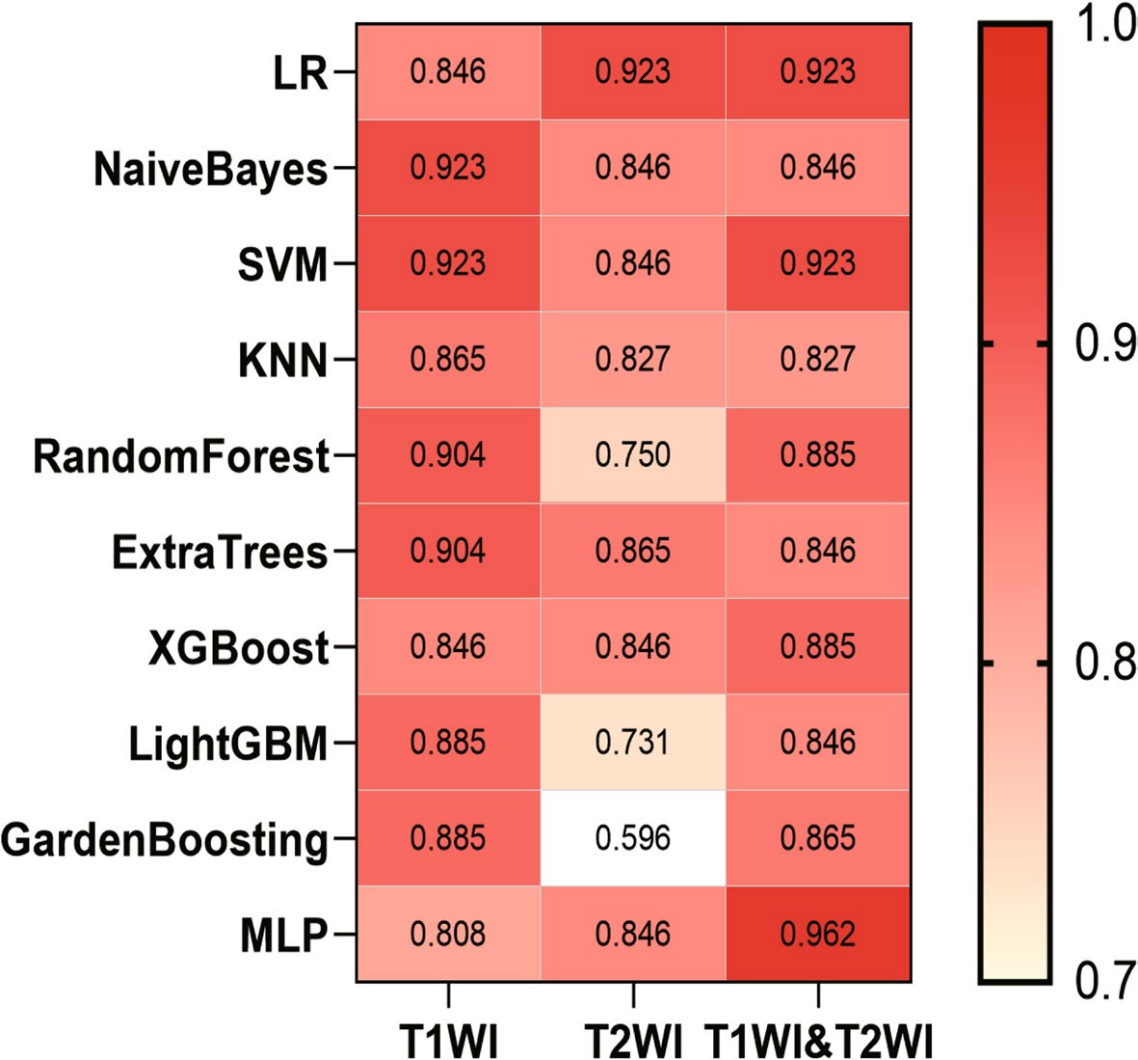
The heatmap of AUC of ten classifiers constructed with different data. A darker color indicates a higher AUC

**Fig. 8:**
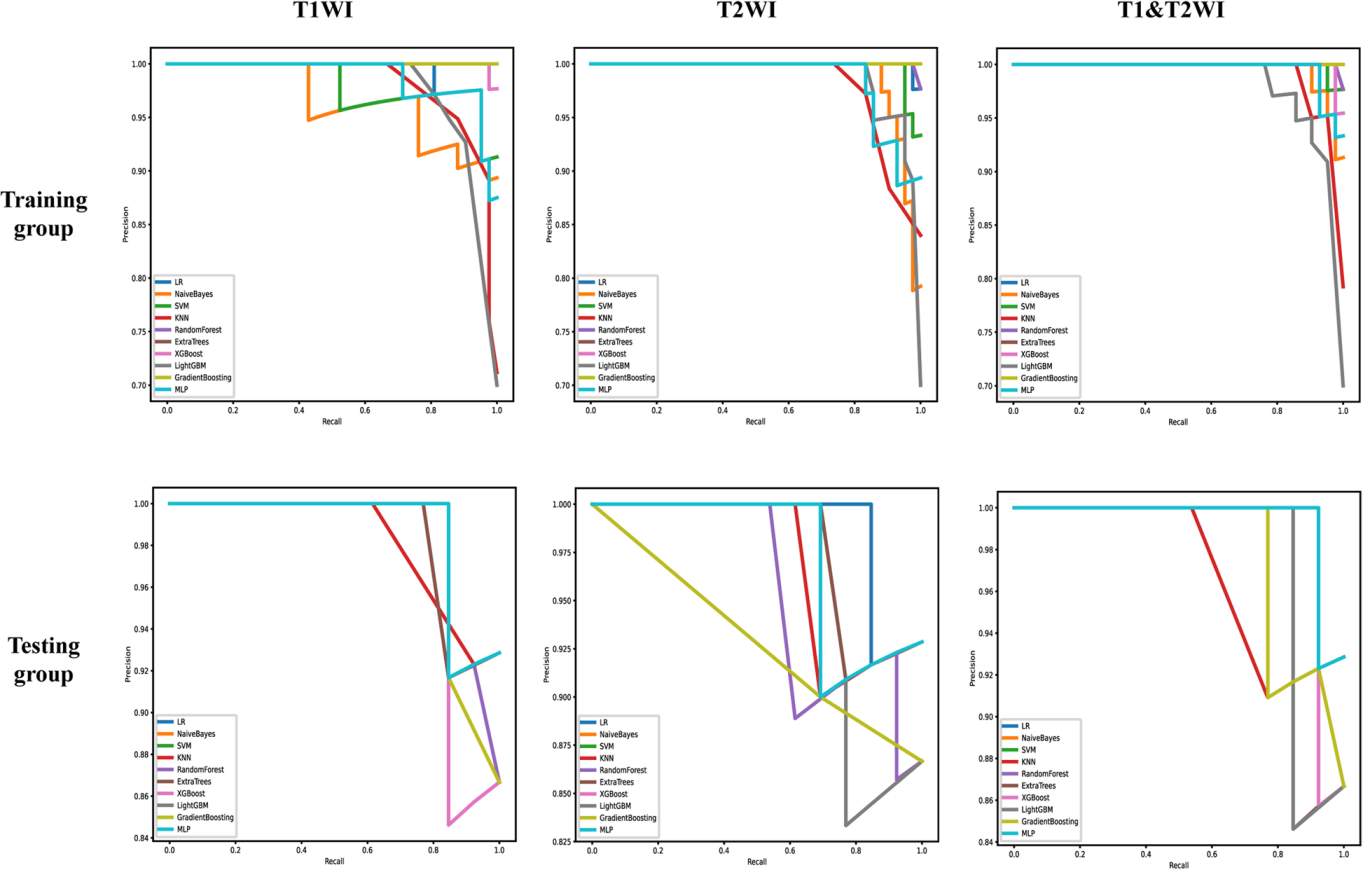
The precision-recall curve of different diagnostic models in the training set and testing set. T1WI (left), T2WI (middle), and T1&T2WI (right). The x-axis represents recall, which indicates how many of the original positive samples were predicted correctly. The y-axis represents precision, which indicates how many of the samples predicted to be positive are positive samples

**Fig. 9:**
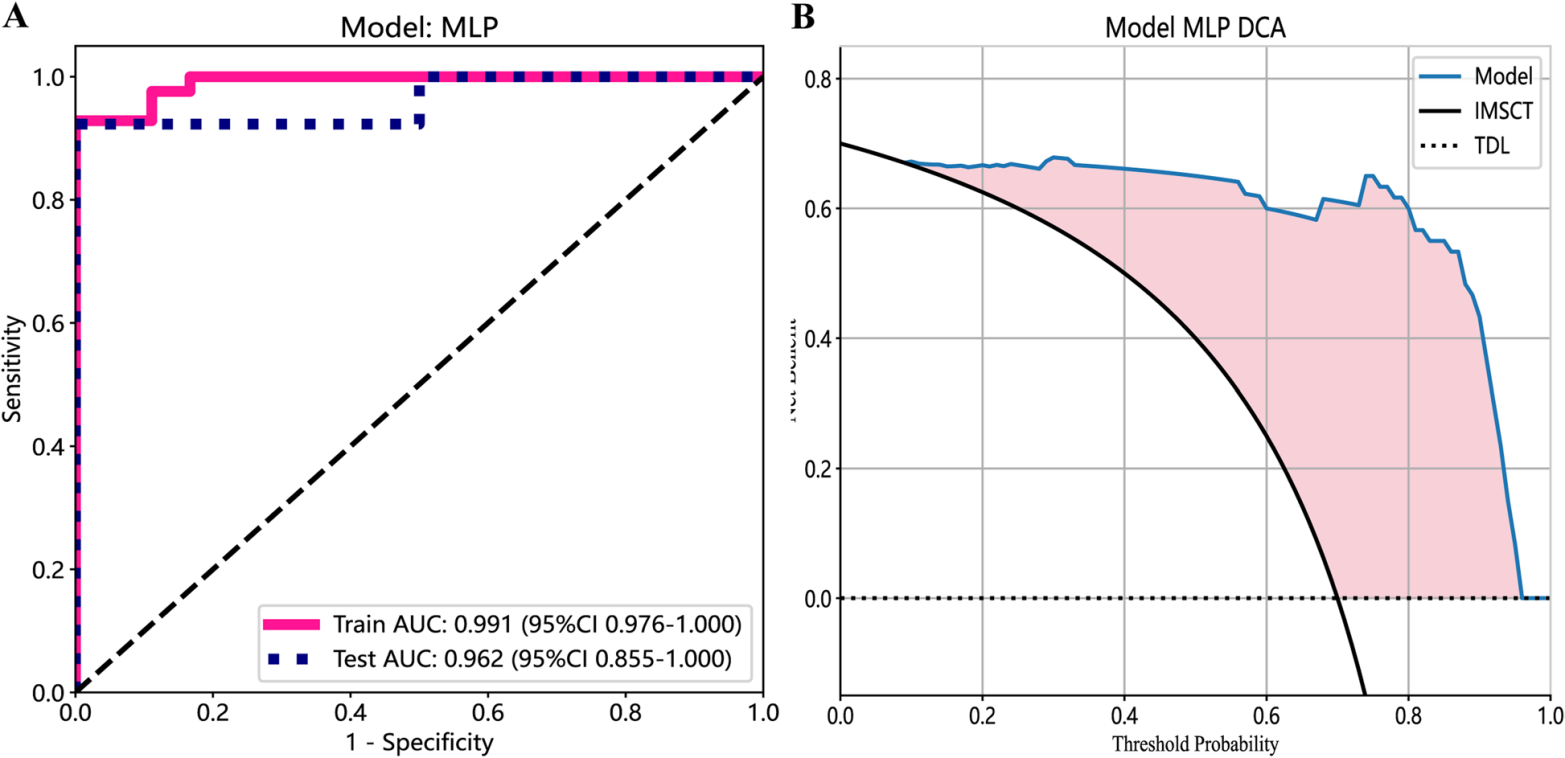
ROC curves (**A**) and DCA curves (**B**) of the MLP network classifier

Ten prediction models were generated using ten different classifiers across either or both MRI sequences, as shown in Fig. 6. The AUC values of these 30 models are depicted in a heatmap diagram in Fig. 7, where darker shades represent higher AUC values. The MLP (Multi-Layer Perceptron) model, which integrates both imaging sequences, achieved the highest AUC (Fig. 7). While DeLong's test indicated that the MLP two-sequence model's advantage over the other models was not statistically significant (*p* > 0.05, Supplementary Table 2), the MLP two-sequence model remained the most effective, as indicated by Precision-Recall curves (Fig. 8), Confusion Matrices (Supplementary Fig. 1), and its highest F1-score and accuracy (Fig. 9). For the training set, the model showed an AUC of 0.991, sensitivity of 92.3%, accuracy of 95.0%, and an F1-score of 96.3%. In the testing set, it achieved an AUC of 0.962, sensitivity of 92.3%, accuracy of 93.3%, Youden's Index of 0.52, specificity of 68.1%, and an F1-score of 96.0%.

### Performance superiority: radiomics model against radiologists' models

**Fig. 10:**
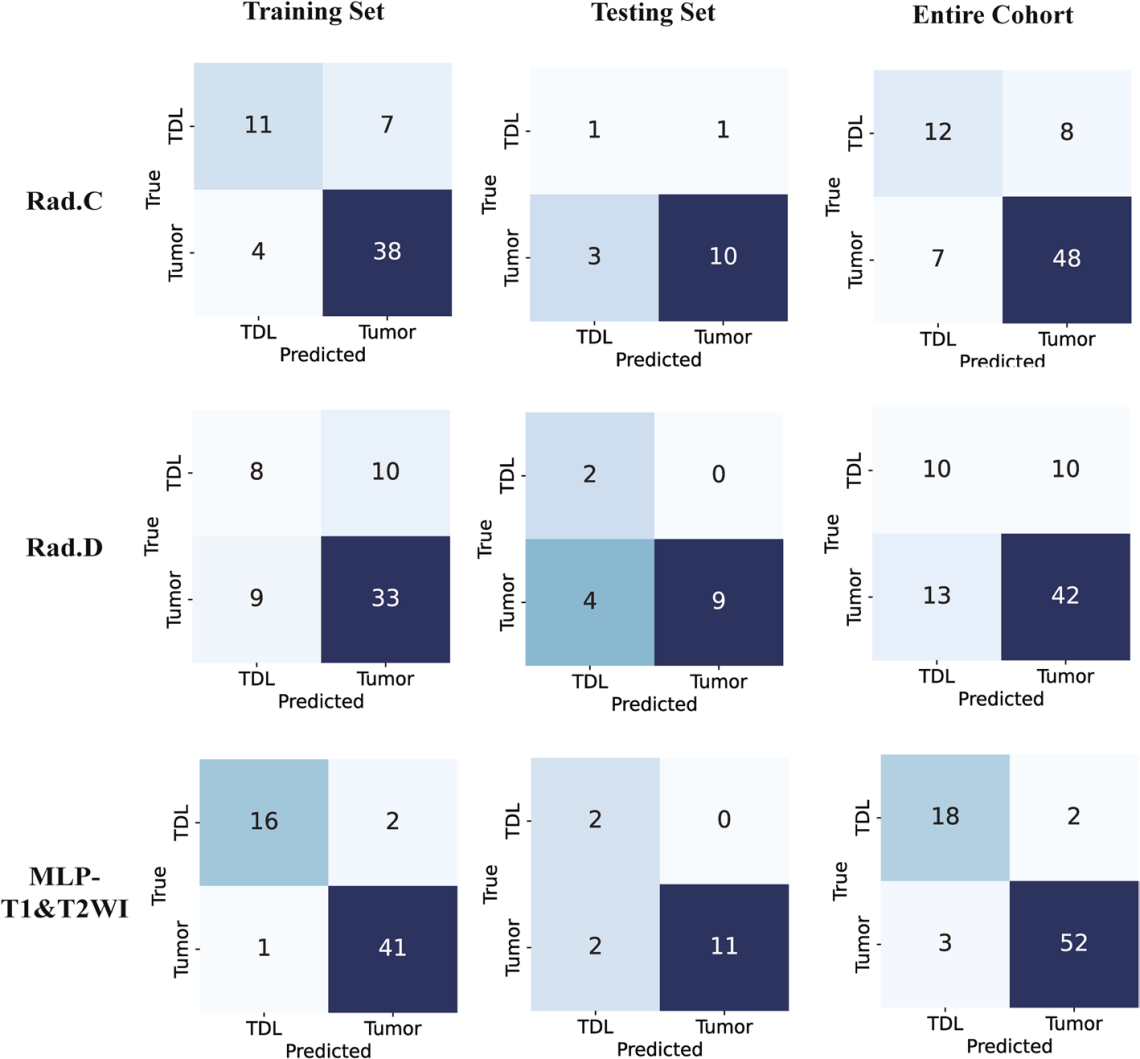
Confusion matrices of the radiologists and MLP two-sequence model in training set, testing set, and the entire cohort

We further opted to employ the MLP two-sequence radiomics model for comparison with the radiologists' diagnoses. First, Kappa analysis determined the good diagnostic agreement between two radiologists, 0.60 to 0.70 in the training group, and 0.70 to 0.75 in the testing group (Supplementary Table 3). DeLong test revealed significant differences favoring the radiomics model over the radiologists' models in the training set and complete cohort (*p* < 0.05, Table 2 and Fig. 10).

**Table 2:** Diagnostic performance of the radiologists and radiomics model

	Training Group			Testing Group			Entire Cohort		
	Rad. C	Rad. D	MLP	Rad. C	Rad. D	MLP	Rad. C	Rad. D	MLP
AUC	0.758	0.615	0.991	0.635	0.846	0.962	0.736	0.632	0.975
Accuracy	0.817	0.683	0.950	0.733	0.733	0.933	0.800	0.693	0.933
F1-score	0.667	0.457	0.963	0.333	0.500	0.96	0.615	0.465	0.878
Sensitivity	0.611	0.444	0.929	0.500	1.000	0.923	0.600	0.500	0.900
Specificity	0.905	0.786	1.000	0.769	0.692	1.000	0.873	0.763	0.945

## Discussion

We developed an MLP two-sequence model, an MRI-based radiomics model, with high efficacy in differentiating IMSCT and scTDL. The superior accuracy, specificity and sensitivity of this model compared to radiologists underscore its potential clinical value. This is the first study employing radiomics models to forecast the diagnosis of intramedullary lesions such as tumors and demyelinating diseases.

MRI represents the key diagnostic assessment in neurological disorders including IMSCT and scTDL. Intramedullary tumors, such as gliomas, and scTDL can appear very similar on MRI, with indistinguishable T1 and T2 signal characteristics. Previous studies have highlighted significant differences between these two lesions in brain in terms of Fractional Anisotropy (FA) and Apparent Diffusion Coefficient (ADC). FA is a measure used in diffusion tensor imaging (DTI) to assess the directionality of water diffusion in tissues, providing insights into the structural integrity of the tissue. ADC, on the other hand, reflects the magnitude of water diffusion, indicating tissue cellularity and integrity. Ducreux et al. [24] observed an average FA value of 0.48 (± 0.02) in five cases of cervical spinal cord astrocytoma, whereas Renoux et al. [25] found an average FA value of 0.588 in inflammatory lesions. Another study reported average FA values of 0.232 (± 0.07) for tumors and 0.39 (± 0.111) for intracranial TDL [26]. These reports indicated that FA tends to be lower in the neoplastic lesion in comparison with the demyelinating insults. ADC values have also been utilized to differentiate between tumors and demyelinating lesions. Marc and his team developed a multi-variable model that includes ADC values, demonstrating an excellent discriminative power (AUC = 0.954–0.986) in distinguishing TDL and glioma [27].

Further efforts to distinguish TDL from tumors have also employed other imaging techniques. For instance, Dae's research highlights that the hypoattenuation observed in computerized tomography (CT) for MR-enhanced lesions offers a high degree of specificity for differentiating between intracranial tumors and TDL [28]. For TDL cases, both MR-enhancing and non-enhancing components showed lower CT attenuation compared to the gray matter in the cortex and basal ganglia. Although tumors frequently exhibited CT hypoattenuation as well, this feature was not present in the MR-enhancing sections [28]. In addition, Satoko et al. employed contrast-enhanced MRI and methionine positron emission tomography (MET-PET) to distinguish brain TDL from tumors, finding that TDL displayed hypometabolism on MET-PET and incomplete ring enhancements on MRI [29]. Further efforts to distinguish TDL from glioma have involved dynamic contrast-enhanced perfusion MRI to assess cerebral blood volume (CBV) and flow (CBF), with demyelinating lesions showing decreased CBV and CBF [30]. Despite these advances, research specifically targeting the differentiation of scTDL from IMSCT is still scarce.

FA and ADC, while useful, have limitations in spinal cord imaging, particularly due to DTI’s lower spatial resolution and susceptibility to artifacts [31]. Our study addresses this by using standard T1- and T2-weighted MRI sequences combined with radiomics features such as shape, texture, and intensity. This approach avoids the technical challenges associated with DTI and advanced imaging techniques, making it more practical for widespread clinical use. Unlike MET-PET, which require specialized equipment, our model can be easily integrated into clinical workflows using conventional MRI, making it a cost-effective and scalable solution for improving diagnostic accuracy in spinal cord lesions.

Radiomics provides a promising approach for characterizing tissue properties using regular CT or MRI imaging. By extracting quantitative features that are undetectable through visual evaluation, radiomics provides an advanced diagnostic method and has been implemented in conditions such as fatty liver and pulmonary nodule, etc. [32, 33]. In the present study, we employed a radiomics method to generate a predictive diagnosis model by analyzing both T1 and T2 MRI images from 75 IMSCT and scTDL individuals. A serial of 9 features including three first-order statistical features, three 3D shape features, two GLSZM features, and one GLDM feature were identified. An MLP model using both sequences were then outstood from 30 models, showing the highest efficacy in differentiating these two lesions.

The finding that the MLP two-sequence model outperformed two junior radiologists is particularly noteworthy as it highlights the potential for this model to assist less experienced clinicians in making more accurate diagnoses. Given the subtle and overlapping imaging characteristics of IMSCT and scTDL, junior radiologists with limited experience in complex spinal cord lesions may struggle to differentiate between these conditions. The model’s ability to surpass their diagnostic accuracy underscores its value as a decision-support tool that can bridge the gap between less experienced and more seasoned clinicians, thereby enhancing diagnostic consistency across different experience levels. Moreover, we used DCA to measure the model's net benefit across various potential risk thresholds. This approach allowed us to examine the effects of different risk thresholds on decision-making. The decision curves demonstrated that employing the prediction model for diagnosing intramedullary conditions proved more advantageous than either universally treating all patients as IMSCT or treating all as scTDL. These results highlight the clinical value of the model, particularly in improving diagnostic accuracy, enhancing decision-making, and optimizing patient management in settings where diagnostic expertise may vary.

The present study developed a predictive model using MRI-based radiomics and MLP classifier that demonstrated high accuracy in distinguishing IMSCT from scTDL. This model’s efficacy in surpassing the diagnostic performance of junior radiologists highlights its potential utility as a non-invasive diagnostic tool for both radiologists and clinicians. Misdiagnosis between these conditions can lead to serious clinical consequences, including unnecessary surgical interventions and complications, as emphasized by previous studies. Clinically, the model provides value not only by supporting radiologists with a more objective, reproducible, and quantitative assessment of imaging features that are difficult to interpret visually but also by assisting clinicians in making informed decisions on patient management.

In clinical practice, this model can be integrated seamlessly into the current diagnostic workflow without the need for significant changes in infrastructure. For example, after routine MRI scans are performed, radiologists can use this model to process the imaging data and generate a radiomics-based diagnostic prediction. Radiologists can then compare the model’s results with their own visual assessments, using the model as a second opinion or as a confirmation in cases where the diagnosis is uncertain or ambiguous. Clinicians, particularly those involved in the treatment of spinal cord lesions, can also use the model’s output to guide clinical decision-making. For instance, in treatment planning, a clear distinction between IMSCT and scTDL is crucial as it dictates very different treatment paths—surgery for IMSCT and medical management for scTDL. However, if relying on CSF diagnosis, the process is invasive and often requires a long waiting period for results. By integrating the model’s results into multidisciplinary discussions between radiologists, neurologists, and surgeons, clinicians can make more informed decisions, improving the accuracy of diagnoses and reducing unnecessary surgical interventions.

Despite these promising results, our study has several limitations. First, the retrospective design may introduce selection bias. Secondly, the MRI images collected in this study were captured using 2 different machines, resulting in potential inconsistencies in data quality. To address this limitation, we standardized imaging procedures and performed ICC analysis to minimize variations across different imaging acquisitions and enhance feature reproducibility [34]. Finally, the dataset was retrospectively compiled from a single center, limiting the diversity of the patient population and thus hinder the universality of our findings.

## Conclusion

In this retrospective study, a predictive model based on two-sequence MRI radiomics features and using an MLP classifier showed high efficacy in distinguishing scTDL and IMSCT. Its diagnostic performance was comparable to that of junior radiologists. These findings support the potential for multicenter, prospective studies with larger patient cohorts to further validate this model's clinical utility.

## Supplementary Information

Supplementary Material 1.

Supplementary Material 2.

## Data Availability

The source code supporting the conclusions of this article is available in the Zenodo repository, accessible via (https://doi.org/10.5281/zenodo.11499435) (https://doi.org/10.5281/zenodo.11499435). The repository ensures open access to the code under the Creative Commons Attribution License, which permits unrestricted use, distribution, and reproduction in any medium, provided the original work is properly cited. Data supporting the findings of this study are not publicly available due to restrictions related to Zhongda Hospital Neurosurgery Department data governance. Further inquiries can be directed to the corresponding author.

## Abbreviations

IMSCT: Intramedullary Spinal Cord Tumor
scTDL: spinal cord Tumefactive Demyelinating Lesion
LR: Logistic Regression
NaiveBayes: Naive Bayes
SVM: Support Vector Machine
KNN: K Nearest Neighbors
RF: Random Forest
ExtraTrees: Extra Trees
XGBoost: eXtreme Gradient Boosting
LightGBM: Light Gradient Boosting Machine
GradientBoosting: Gradient Boosting
MLP: Multi-Layer Perceptron
AUC: Area Under the Curve
MRI: Magnetic Resonance Imaging
VOI: Volume of Interest
ICC: Intraclass Correlation Coefficient
GLCM: Gray Level Co-occurrence Matrix
GLRLM: Gray Level Run Length Matrix
GLSZM: Gray Level Size Zone Matrix
NGTDM: Neighbouring Gray Tone Difference Matrix
LASSO: Least Absolute Shrinkage and Selection Operator
ROC: Receiver Operating Characteristic
DTI: Diffusion tensor imaging
CT: Computerized Tomography
MET-PET: Methionine Positron Emission Tomography
CBV: Cerebral Blood Volume
CBF: Cerebral Blood Flow
