# Supplementary material for: Establishment of an MRI-based radiomics model for distinguishing between intramedullary spinal cord tumor and tumefactive demyelinating lesion
Source: BMC Med Imaging. 2024 Nov 21;24:317. doi: 10.1186/s12880-024-01499-8 (PMC11583559; doi:10.1186/s12880-024-01499-8)
Supplement: Supplementary file 1 — Supplementary Material 1. [file 12880_2024_1499_MOESM1_ESM.docx]

**Table.1** MRI Parameters of Selected Sequences

| Parameters | Philips Ingenia 3.0T | | Siemens Verio 3.0 T | |
| --- | --- | --- | --- | --- |
| Cervical vertebra | T1WI | T2WI | T1WI | T2WI |
| Repetition time(ms) | 470 | 2500 | 1600 | 2480 |
| Echo time(ms) | 474 | 85 | 9.4 | 103 |
| FOV(mm^2^) | 220×255 | 220×258 | 260×260 | 260×260 |
| Matrix | 244×253 | 244×230 | 192×320 | 257×384 |
| Slice thickness(mm) | 3 | 3 | 4 | 4 |
| Slice gap(mm) | 0.5 | 0.5 | 0.4 | 0.4 |
| Thoracic vertebra |  |  |  |  |
| Repetition time(ms) | 450 | 2500 | 1600 | 2400 |
| Echo time(ms) | 12 | 90 | 8.6 | 101 |
| FOV(mm^2^) | 240×360 | 240×360 | 300×300 | 300×300 |
| Matrix | 252×255 | 252×256 | 179×256 | 288×384 |
| Slice thickness(mm) | 3 | 3 | 4 | 4 |
| Slice gap(mm) | 0.5 | 0.5 | 0.4 | 0.4 |
| Lumbar vertebra |  |  |  |  |
| Repetition time(ms) | 684 | 2500 | 1500 | 2400 |
| Echo time(ms) | 16 | 85 | 8.6 | 1.6 |
| FOV(mm^2^) | 220×288 | 220×287 | 320×320 | 320×320 |
| Matrix | 316×249 | 244×244 | 179×256 | 307×384 |
| Slice thickness(mm) | 3 | 3 | 4 | 4 |
| Slice gap(mm) | 0.5 | 0.5 | 0.4 | 0.4 |

**Table.2** Absolute P values of the MLP-T1&T2WI model vs other 29 models

|  | Training | Testing |
| --- | --- | --- |
| MLP-T1&T2WI vs LR-T1WI | 0.3998 | 0.2076 |
| MLP-T1&T2WI vs NaiveBayes -T1WI | 0.0925 | 0.4795 |
| MLP-T1&T2WI vs SVM-T1WI | 0.2962 | 0.4795 |
| MLP-T1&T2WI vs KNN-T1WI | 0.0496 | 0.1545 |
| MLP-T1&T2WI vs RandomForest -T1WI | 0.2262 | 0.3428 |
| MLP-T1&T2WI vs ExtraTrees -T1WI | 0.2262 | 0.4171 |
| MLP-T1&T2WI vs XGBoost -T1WI | 0.2754 | 0.2076 |
| MLP-T1&T2WI vs LightGBM -T1WI | 0.0333 | 0.1944 |
| MLP-T1&T2WI vs GradientBoosting -T1WI | 0.2262 | 0.1944 |
| MLP-T1&T2WI vs MLP -T1WI | 0.2399 | 0.4795 |
| MLP-T1&T2WI vs LR-T2WI | 0.2771 | 0.4795 |
| MLP-T1&T2WI vs NaiveBayes -T2WI | 0.2295 | 0.3763 |
| MLP-T1&T2WI vs SVM-T2WI | 0.6427 | 0.3763 |
| MLP-T1&T2WI vs KNN-T2WI | 0.0444 | 0.3621 |
| MLP-T1&T2WI vs RandomForest -T2WI | 0.2458 | 0.1896 |
| MLP-T1&T2WI vs ExtraTrees -T2WI | 0.2262 | 0.3817 |
| MLP-T1&T2WI vs XGBoost -T2WI | 0.2262 | 0.3763 |
| MLP-T1&T2WI vs LightGBM -T2WI | 0.1935 | 0.0321 |
| MLP-T1&T2WI vs GradientBoosting -T2WI | 0.2262 | 0.0969 |
| MLP-T1&T2WI vs MLP -T2WI | 0.0963 | 0.3763 |
| MLP-T1&T2WI vs LR-T1&T2WI | 0.2218 | 0.4795 |
| MLP-T1&T2WI vs NaiveBayes -T1&T2WI | 0.8413 | 0.1088 |
| MLP-T1&T2WI vs SVM-T1&T2WI | 0.2487 | 0.4795 |
| MLP-T1&T2WI vs KNN-T1&T2WI | 0.0849 | 0.3531 |
| MLP-T1&T2WI vs RandomForest -T1&T2WI | 0.2217 | 0.1396 |
| MLP-T1&T2WI vs ExtraTrees -T1WI | 0.2262 | 0.2076 |
| MLP-T1&T2WI vs XGBoost -T1WI | 0.3528 | 0.1396 |
| MLP-T1&T2WI vs LightGBM -T1WI | 0.0849 | 0.2076 |
| MLP-T1&T2WI vs GradientBoosting -T1WI | 0.2262 | 0.2207 |

**Table.3** Kappa values between two radiologists

|  | **Training group** |  | **Testing group** |  | **Entire Cohort** |  |
| --- | --- | --- | --- | --- | --- | --- |
|  | **Kappa value** | **P** | **Kappa value** | **P** | **Kappa value** | **P** |
| Rad. C vs Rad. D | 0.660 | ＜0.001 | 0.706 | 0.004 | 0.670 | ＜0.001 |
