# Supplementary figures and images for: Establishment of an MRI-based radiomics model for distinguishing between intramedullary spinal cord tumor and tumefactive demyelinating lesion
Source: BMC Med Imaging. 2024 Nov 21;24:317. doi: 10.1186/s12880-024-01499-8 (PMC11583559; doi:10.1186/s12880-024-01499-8)

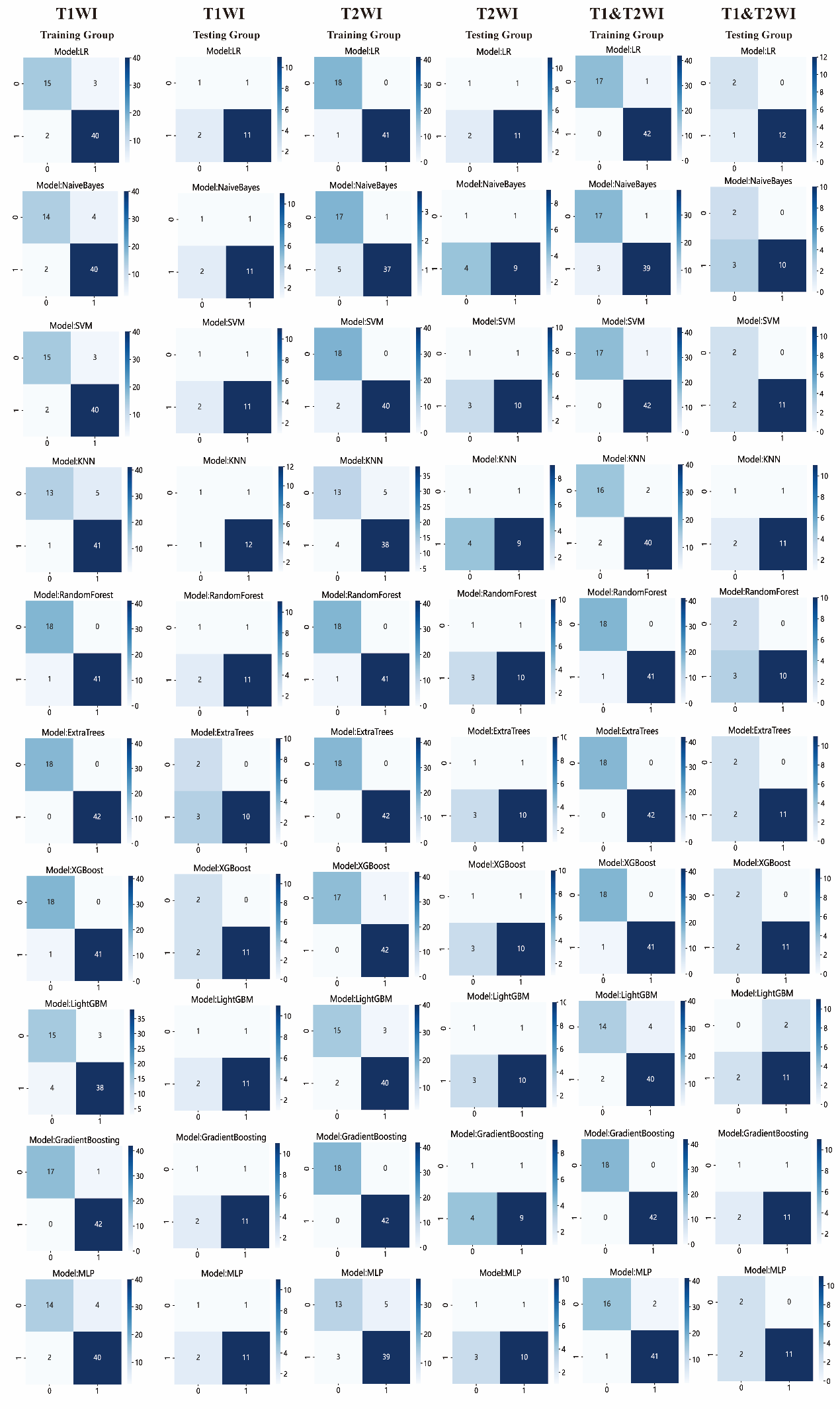


**Supplementary Fig. 1** Confusion Matrices of 30 models in training and testing groups.

Supplement: Supplementary file 2 — Supplementary Material 2. [file 12880_2024_1499_MOESM2_ESM.docx]
